# Effects of exercise anticipation on cardiorespiratory coherence

**DOI:** 10.14814/phy2.15381

**Published:** 2022-07-26

**Authors:** Aditya Koppula, Ram Reddy Barra, Kousik Sarathy Sridharan

**Affiliations:** ^1^ Department of Biomedical Engineering, Neurotech Lab Indian Institute of Technology Hyderabad India; ^2^ Department of Physiology Apollo Institute of Medical Sciences and Research Hyderabad India

**Keywords:** autonomic asymmetry, cardiorespiratory coupling, coherence, exercise anticipation, heart rate variability, respiratory sinus arrhythmia

## Abstract

In this study, we explored the role of feedforward mechanisms in triggering cardiorespiratory adjustments before the onset of exercise. To isolate the feedforward aspects, we examined the effect of exercise anticipation on cardiorespiratory coherence. Twenty‐nine healthy males (age = 18.8 [0.96] years) were subjected to bicycle (BE) and handgrip exercise (H) at two different intensities, viz.*,* low and high. Bicycle exercise was performed in a unilateral (left‐ and right‐sided) or bilateral mode, whereas handgrip was performed only in a unilateral mode. Single‐lead ECG and respiratory rhythm, measured in the 5 min of anticipation phase before the onset of exercise, were used for analysis. Coherence was computed between ECG‐derived instantaneous heart rate and respiratory signal. Average coherence in the high‐frequency band (0.15–0.4 Hz) was used to estimate respiratory sinus arrhythmia (RSA). We found that coherence decreased with the anticipation of exercise relative to baseline (baseline = 0.54 [0.16], BE = 0.41 [0.12], *H* = 0.39 [0.12], *p* < 0.001). The decrease was greater for high intensity exercise (low = 0.42 [0.11], high = 0.37 [0.1], *p* < 0.001). The fall of coherence with intensity was stronger for bicycle exercise (BE: low = 0.44 [0.12], high = 0.37 [0.12], H: low = 0.4 [0.12], high = 0.37 [0.12], *p* = 0.00433). The expectation of bilateral exercise resulted in lower coherence compared to unilateral exercise (right‐sided = 0.45 [0.16], left‐sided = 0.4 [0.16], bilateral = 0.36 [0.15], unilateral vs. bilateral: *p* < 0.001), and the left‐sided exercise had lower coherence compared to that of the right (left‐sided vs. right‐sided: *p* = 0.00925). Handgrip exercise showed similar trend (right‐sided = 0.4 [0.15], left‐sided = 0.37 [0.14], *p* = 0.0056). In conclusion, feedforward RSA adjustments in anticipation of exercise covaried with subsequent exercise‐related features like intensity, muscle mass (unilateral vs. bilateral), and the exercise side (left vs. right). The left versus the right difference in coherence indicates autonomic asymmetry. Feedforward changes in RSA are like those seen during actual exercise and might facilitate the rapid phase transition between rest and exercise.

## Graphical abstract

Cardiorespiratory coupling (CRC), measured as coherence, decreased with the expectation of exercise and the decrease scaled with the type, intensity, and laterality of the anticipated exercise. Occurrence of these changes before the onset of exercise and predictable covariation with the exercise settings suggests that patterned visceral adjustments are integrated into motor planning and enable a rapid change in physiological state of the organism. Decreased CRC in these settings can have cascading effects on respiratory gas exchange and workload on the heart with implications in health and disease states.

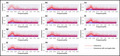

## 1 INTRODUCTION

Exercise anticipation evokes a multidimensional set of cognitive, somatic, and visceral adjustments (Van Paridon et al., 2017), occurring in a feedforward manner (Secher, 2007) during the transition between rest and actual physical exercise. Cognitive changes include attentional, arousal, and emotional responses (Van Paridon et al., 2017); somatic changes include tensing of skeletal muscles; visceral changes include metabolic and autonomic responses (Tobin et al., 1986). Such feedforward mechanisms play an essential role in the rapid organization of integrated somatic‐visceral response in expectation of the initiation of voluntary movements. Somatic‐visceral output includes descending motor commands that cause movements, supporting metabolic‐Endocrine and cardiorespiratory adjustments (Obrist, 1981). Changes in the integrated cardiorespiratory output, termed cardiorespiratory coupling (CRC), are pre‐requisites for initiating and maintaining the exercise state. Krogh and Lindhard (1913) noted striking changes in ventilation and heart rate in expectation of a heavy exercise load similar to what is seen with actual exercise. A similar increase in ventilation was observed in subjects who were asked to imagine an uphill cycling (Thornton et al., 2001). It was proposed that such feedforward adjustments buffered a sudden increase in partial pressure of carbon dioxide and a fall in partial pressure of oxygen, in the arterial blood, with the onset of exercise (Krogh & Lindhard, 1913). These anticipatory adjustments are mainly due to (a) Central command from motor cortical centers to brain stem cardio‐respiratory centers and (b) Afferent input from exercising muscles (Krogh & Lindhard, 1913; Paterson, 2014). Thus, though cardiac and respiratory rhythms are known to be altered in the anticipation of exercise, in a load‐dependent manner, the accompanying adjustment of CRC and its physiological purpose are not entirely understood. Therefore, we focused on CRC changes inherent in somatic‐visceral response to exercise anticipation.

Cardiorespiratory coupling reflects the bidirectional nature of cardiorespiratory interaction and it encompasses several distinct phenomena, viz., respiratory sinus arrhythmia (RSA), cardiorespiratory phase synchronization (CRPS; Bartsch et al., 2012), and cardioventilatory coupling (CVC; Dick et al., 2014). RSA is defined as the modulation of heart rate by respiratory rhythm, that is, heart rate acceleration during inspiration and deceleration during expiration (Garcia et al., 2013). CRPS is the occurrence of R‐peaks at particular respiratory cycle phases and indicates phase locking between the two rhythms (Schäfer et al., 1998, 1999). Respiration influences not only the heart rate but also the blood pressure (Hering‐Traube waves). In contrast to the above, where respiration influenced cardiovascular rhythms, CVC is the influence of blood pressure on the inspiration onset (Coleman, 1920; Friedman et al., 2012; Larsen & Galletly, 1999). CRC is thought to occur at the level of brain stem cardio‐respiratory central pattern generators (Garcia et al., 2013). The physiological purpose of CRC is a subject of active investigation, and it has been variously proposed that it improves the efficiency of gas exchange (Hayano & Yasuma, 2003) in the lungs or that it minimizes the work done by the heart while maintaining the blood gas concentrations in the physiological range (Ben‐Tal et al., 2012). From a teleological perspective, we conjecture that changes in CRC in the anticipation phase facilitate the phase transition between rest and exercise and predict the subsequent motor performance. As exercise anticipation, by definition, minimizes the role of movement‐related afferent input from the muscles, central commands might be responsible for the cardio‐respiratory coupling changes during the anticipation phase. Also, by exercising the left and right limbs independently, the existence of autonomic asymmetry (Ahern et al., 2001; Craig, 2005; Lane et al., 2009) in the context of exercise, on the lines of cerebral asymmetry for higher functions, can also be explored.

Several methods are used to quantify various aspects of CRC (Schulz et al., 2013; Tonu et al., 2020). In this study, we used *coherence* between instantaneous heart rate and respiratory rhythm, averaged in the high‐frequency band of Heart rate variability (HRV), that is, 0.15–0.4 Hz, as a measure of RSA, to characterize CRC changes induced by anticipation of exercise. We also computed frequency domain HRV metrics (Shaffer et al., 2017), that is, *total power* (TP), a measure of overall heart rate variability, *high‐frequency power in normalized units* (*HFnu*), a measure of vagal modulation of heart rate (Shaffer et al., 2017) and *LF/HF ratio*, an index of sympathovagal balance (Shaffer et al., 2017).

The current study explores cardiorespiratory‐autonomic changes in the anticipatory period of two specific dynamic and static exercise types, that is, bicycling, and isometric handgrip. We examined the effect of mere expectation of exercise on coherence. We formulated two hypotheses: (1) Anticipatory period changes in coherence are related to the type and intensity of exercise and muscle mass involved in the subsequent physical activity. (2) Differential response of coherence to the left‐ and right‐sided exercise suggestive of asymmetry in the autonomic nervous system. Consistent variation of anticipatory period coherence with exercise‐related variables would have the following implications: (a) Internal models of movement in the motor system include cardiorespiratory‐autonomic components. (b) Pre‐movement phase coherence is predictive of subsequent motor performance in health and disease.

## 2 METHODS

### 2.1 Participants

Thirty healthy students from Gandhi medical college, Hyderabad, participated in the study. The study was approved by the Institutional ethical committee, Gandhi medical college (Rc.No.IEC/GMC/2017/XXXVIII). The study conformed to the standards set by the Declaration of Helsinki. Informed consent was taken from each participant. Male participants in the age group of 18–25 years were included, and those with congenital heart disease, rheumatic heart disease, chronic respiratory disease, neuromuscular, and musculoskeletal ailments, that precluded the performance of exercise were excluded from the study. Of the 30 subjects, one had pulses trigeminus when the ECG was recorded and was excluded (Figure S1). The final study cohort thus had 29 participants with the details listed in Table 1.

**TABLE 1:** Participant details

Measure	Mean (SD)
Age (years)	18.83 (0.91)
Height (cm)	169.93 (5.77)
Weight (kg)	64.28 (13.93)
Age estimated maximum heart rate (220‐age) (beats/min)	201.2 (0.96)
Baseline heart rate (beats/min)	85.25 (12.04)
Total number of participants: 29 (males = 29, females = 0)	

### 2.2 Data acquisition

The ECG and respiratory rhythm were acquired by ADInstruments® Powerlab‐15 T system and LABCHART8 software. ECG was recorded in a single lead configuration with electrode placement customized to facilitate recordings with minimal motion artifacts during exercise. An active electrode was applied over the left 5th intercostal space below the nipple on the position of the apex beat. Referring electrode was applied in the right supraclavicular fossa. The ground electrode was applied on the right side of the chest over the right 5th intercostal space below the nipple (Figure S2). The respiratory rhythm was recorded using Pneumotrace, a piezo respiratory belt transducer (MLT1132) tied around the abdomen. The time‐series data were sampled at 1 kHz.

### 2.3 Experimental paradigm

**FIGURE 1:**
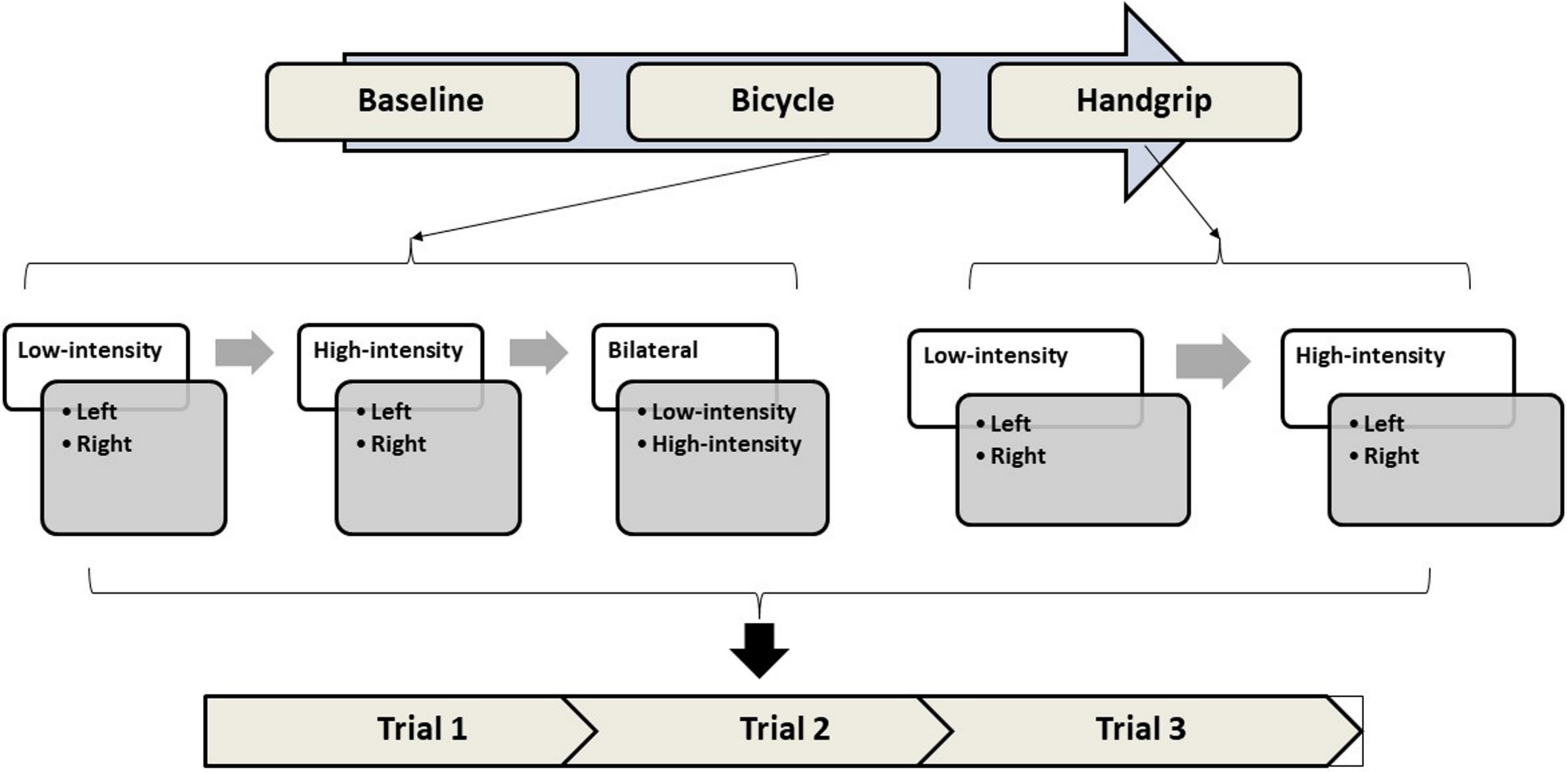
Experimental paradigm. Exercises are performed in an invariant order for all the participants as shown in the Figure 1. For each of the two types of exercise, that is, Bicycling and handgrip, low intensity was followed by high intensity. For each intensity, left sided exercise was performed first followed by the right. Bilateral exercise was performed only for bicycle exercise. For each of the factorial combinations of type, intensity and side of exercise, three repetitions/trials were done. Of the three trials, two were real trials and one was sham trial.

Before the recording, participants were instructed to avoid heavy exercise for 3 days; smoking and alcohol for at least 1 week. They were advised to sleep for 8 h on a preceding night and come to the lab in the morning after a light breakfast. After a resting period of 15 min, baseline ECG and respiratory rhythm were recorded for 5 min. Every participant was subjected to bicycle and isometric handgrip exercise at low and high intensity. Each exercise intensity was done in a left‐sided, right‐sided, and bilateral manner for bicycle exercise. In contrast, the handgrip exercise was done with the left hand and the right hand for each level of intensity. Therefore, there were six factorial combinations of intensity and side of exercise for bicycle exercise and four varieties for Handgrip exercise for 10 combinations of type, intensity, and side of exercise. Three trials were done for each of the 10 combinations of exercises mentioned above. There were real trials, in which the anticipation phase was followed by exercise, and sham trials, where they were asked not to perform exercise after the anticipation phase. The real and sham trials are randomized. The duration of each trial of exercise depended on the type of trial. The real trials include 5 min of anticipation and 30 s of exercise. In contrast, sham trials stop at the end of the 5 min of anticipation phase, as there is no exercise following it. The order in which various exercise combinations were performed and the organization of trials in each exercise are shown in Figure 1.

Subjects were told in advance of the exercise's type, intensity, and side before every trial, to induce the effects of anticipation. All the participants underwent training sessions before the actual trials. After the baseline (5‐min resting phase ECG/respiratory signal) recording, participants practiced reaching and maintaining the target intensity of exercise for both the sides and types of activity (bicycle and handgrips). We gave each participant 1–2 practice sessions for every type of exercise. A rest period intervened after completing all the practice trials before actual exercise trials commenced.

The subject was asked to close their eyes at the beginning of each exercise trial. Two minutes after the onset of recording, an auditory cue was played upon which the subject opened their eyes and gazed at the timer displayed on the screen for the next 3 min. At the end of 3 min, participants exercised for 30 s if it was an actual trial, while participants relaxed and waited for the subsequent trial if it was a sham trial. This paradigm was implemented with a LabChart macro. After every exercise, a rest period intervenes until the heart rate returns to the baseline and stays in the baseline range for 1 min to ensure a washout of previous exercise effects and avoid carryover bias. ECG and respiratory signals in the anticipation period prior to the onset of exercise were used for further analysis.

#### 2.3.1 Bicycle exercise

Bicycle exercise was performed on an elliptical trainer bicycle with moving arms (Figure S2), which allows upper limb movements and leg pedaling. Low intensity for Bicycle exercise was defined as the intensity at which 50% of age estimated maximal heart rate was achieved, while it was 70% for the high intensity. Age estimated maximal heart rate was set for every participant by Fox's formula of 220‐Age (years; Sporis et al., 2011). Every participant was instructed to exercise after the second auditory cue, reach the target heart rate as fast as possible, and maintain it for 30 s for all the actual trials. The computer screen displayed the instantaneous heart rate, estimated from the ECG, and the target heart rate. A snapshot of the computer screen showing the participant's visual feedback is shown in supplementary information (Figure S3). The participants were advised to adjust the exercise intensity and match the instantaneous heart rate to the target heart rate. Unilateral exercises involved the movement of both upper and lower limbs on one side alone, whereas bilateral exercises simultaneously involved both the left and right sides.

#### 2.3.2 Hand grip exercise

Isometric handgrip exercise was done by compression of a grip force transducer (MLT004/ST). Low intensity for handgrip exercise was defined as grip force at 35% of maximal grip force and high intensity at 50% of maximal grip force. Maximum grip force was estimated by asking each participant to apply a momentary gripping pressure with maximal effort and taking the highest value from four attempts at 10‐s intervals. Instantaneous grip force and target grip force were displayed on the computer screen (Figure S4), and the participant was instructed to match the instantaneous force with the target force. Unlike the bicycle exercise, the handgrip exercise involved only the left‐sided and right‐sided exercises for each level of intensity. After the second auditory cue, the participant was instructed to compress the transducer to reach the target grip force as fast as possible and maintain it for 30 s for all the actual trials.

### 2.4 Signal processing pipeline

ADInstruments‐SDK‐python toolbox (Hokanson, 2019) was used to import the data from the LabChart, and all the subsequent signal processing was done in Python (Van Rossum & Drake, 2009) using Biosppy (Carreiras et al., 2015), pyhrv (Gomes, 2019), and SciPy packages (Virtanen et al., 2020).

The ECG was analyzed by an inbuilt function *Biosppy.signals.ecg.ecg ()*, from the Biosppy package, which accepted raw, unfiltered ECG as the input and returned instantaneous heart rate and RR‐intervals. The instantaneous heart rate estimated above was interpolated by cubic spline and resampled at 4 Hz to yield a uniformly sampled heart rate time series that was 5 min long. The respiratory rhythm signal was analyzed by another inbuilt function *Biosppy.signals.resp.resp ()*, which accepted raw, unfiltered respiratory signals and returned a filtered version of it as one of the outputs. The raw respiratory signal is bandpassed between 0.1–0.35 Hz using 2nd order linear‐phase Butterworth filter (zero‐phase implementation). This signal was spline interpolated and resampled at 4 Hz to produce a signal of the same length as the interpolated‐resampled heart rate signal.

#### 2.4.1 Cardiorespiratory coherence

Coherence is a frequency‐domain metric that quantifies the linear relationship between two signals/time series. It is a bivariate measure and captures the strength of variation in one time series at the dominant frequencies of another time series. RSA is measured by computing coherence between interpolated‐resampled heart rate time series and respiratory time series (Equation 1). Calculated as above, the metric estimates the power of heart rate variations at the respiratory frequencies. It is a normalized metric, and its values range from 0 to 1. Coherence of 1 indicates that heart rate fluctuations are in complete lockstep with respiration and maximum RSA. In contrast, coherence of 0 means no respiratory‐induced heart rate changes. The closer the coherence value to 1 (high coherence) at the respiratory frequencies, the stronger is the RSA and vice‐versa with coherence values close to 0 (low coherence/weak RSA). We used the coherence method to measure CRC/RSA as it yields a statistically stable estimate with short stretches of data.

(1) $$\begin{array}{c} \mathrm{Cxy}\left(f\right)=\frac{{\left(X{\left(f\right)}^{*}\cdot Y\left(f\right)\right)}^{2}}{\left(X{\left(f\right)}^{*}\cdot X\left(f\right)\right)\cdot \left(Y{\left(f\right)}^{*}\cdot Y\left(f\right)\right)} \end{array}$$

If *x* and *y* are two signals (like heart rate and respiration), *Cxy(f) =* Coherence between *x* and *y* as a function of frequency, *f* = frequency, *X(f)* = FFT (fast fourier transform) of time series *x*, X(f)* = complex conjugate of *X(f), Y(f)* = FFT of time series y, Y(f)* = complex conjugate of *Y(f)*.

**FIGURE 2:**
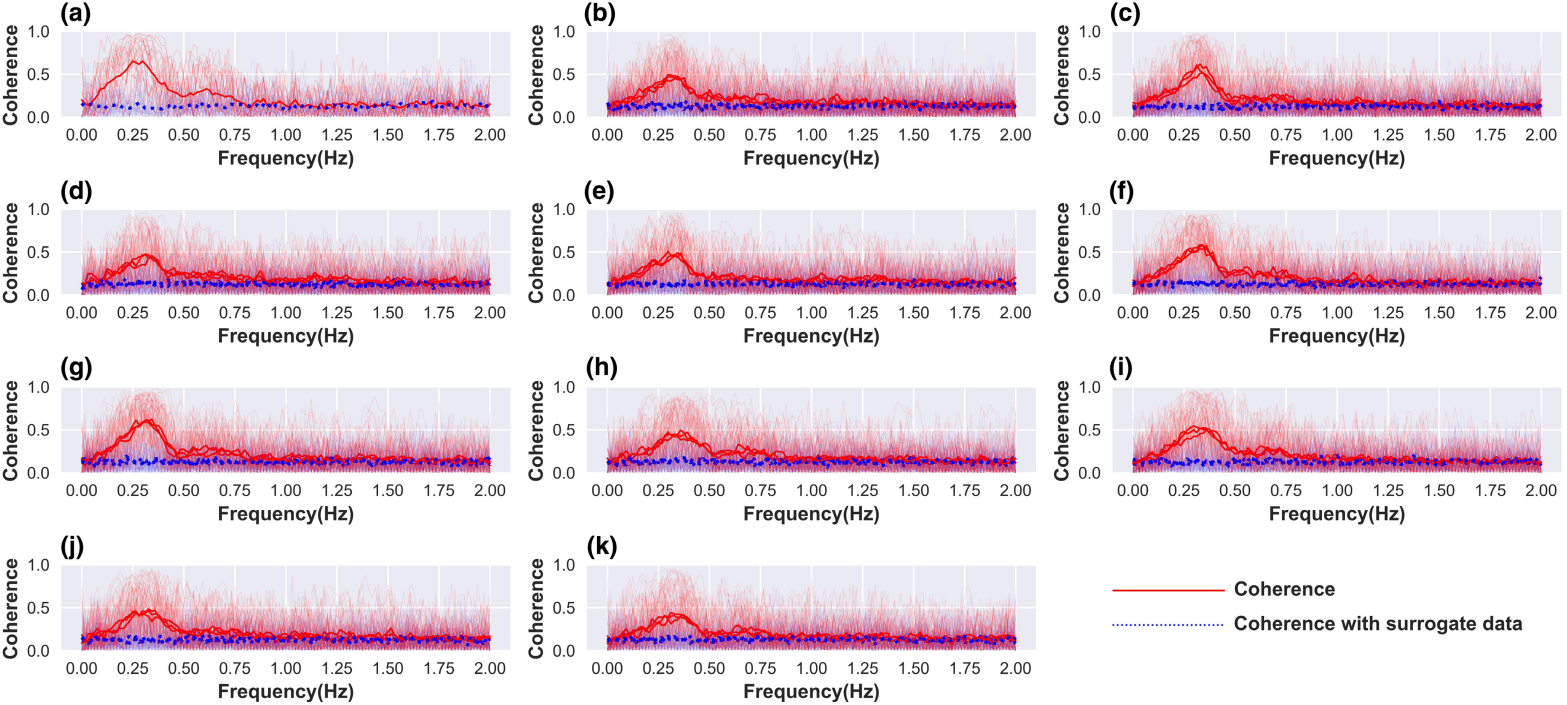
Cardiorespiratory coherence and surrogate data analysis for each exercise condition. Solid curves represent coherence, and the dotted curves represent coherence for surrogate data, created from the same by randomly shuffling the heart rate time series. In health, Coherence plot has a peak in the high frequency region (0.15–0.4 Hz) (3 solid curves, 1 for each trial, in subplots (a–k), that is drastically reduced for shuffled data (3 dotted curves, 1 for each trial, in subplots from (a)–(k). (a) Baseline, (b) handgrip‐low intensity‐left, (c) handgrip‐low intensity‐right, (d) handgrip‐high intensity‐left, (e) handgrip‐high intensity‐right, (f) bicycle‐low intensity‐left, (g) bicycle‐low intensity‐right, (h) bicycle‐high intensity‐left, (i) bicycle‐high intensity‐right, (j) bicycle‐low intensity‐bilateral, (k) bicycle‐high intensity‐bilateral. Translucent curves in each subplot are the individual coherence curves, whereas the bold‐faced curve represents the mean of the same for all the 29 participants.

An inbuilt function *scipy.signals.coherence()*, from the SciPy package, was used to compute coherence as defined in Equation (1). It is based on Welch's method, and we have used the following parameter settings: Hann's window, length of each segment = 256, the overlap between segments = 128, size of fft = 256, and mean detrending. Typical coherence shows a peak/maximum in the high‐frequency region of HRV, that is, 0.15–0.4 Hz (Figure 2), which was averaged to yield a single value for coherence. Surrogate data analysis was used to assess the effect of random fluctuation in heart rate on coherence. We compared the coherence calculated above with that calculated between surrogate heart rate time series and respiratory rhythm. The result was visually inspected to qualitatively assess the non‐random origin of coherence in the high‐frequency region. Surrogate heart rate time series was created by randomly shuffling the interpolated‐resampled heart rate time series (Kabir et al., 2010).

#### 2.4.2 Heart rate variability

The frequency‐domain HRV measures were estimated, from the RR‐intervals, by the Welch method using the function, *fd.welch_psd ()* in the *pyhrv* package(4096‐point FFT, equally sized hamming window and mean detrending). The frequency‐domain HRV metrics extracted were TP, HFnu, and LF/HF ratio. TP is the total area under the power spectral density curve (PSD) between 0–0.4 Hz. It is a measure of overall heart rate variability and a frequency domain equivalent of time‐domain metric SDNN (standard deviation of NN‐ intervals [RR‐intervals]). HF is the area under the PSD curve between 0.15–0.4 Hz, and the LF is similarly computed between 0.05–0.15 Hz. TP was subjected to logarithmic transformation to correct the right skew nature of the data, and used for further analysis (logTP). HFnu was computed by normalizing HF with the sum of LF and HF (LF + HF) as the denominator. Normalization is performed to account for changes in the TP on HF. HFnu measures vagal modulation of heart rate. LF/HF ratio, on the other hand, is a metric of sympathovagal balance and is, elevated in situations provoking sympathetic stimulation of the heart.

### 2.5 Statistical analysis

We used R software for statistical analysis. Linear mixed models were used, as they are the robust methods for analyses of repeated measures data. We modeled the influence of various exercise‐related variables on the outcome measures, Viz., coherence, logTP, HFnu, and LF/HF ratio. We used the *nlme* package for the mixed‐effects modeling.

We reduced the model complexity by aggregating the values of outcome measures at the level of the predictor variable for every participant. For example, to test the effect of the type of exercise, Coherence values were averaged for each level of the predictor variable, that is, baseline, bicycle exercise, and handgrip exercise. Each participant has a single value of coherence for baseline, 18 values for bicycle exercise (2 levels of intensity × 3 levels of sidedness × 3 trials), and 12 values for handgrip exercise (2 levels of intensity × 2 levels of sidedness × 3 trials). Data aggregation is done by averaging all values for bicycle exercise and handgrip exercise to yield a single value of coherence for both types of exercise. Such data aggregation over predictor variables was done before the mixed model analysis. As the levels of sidedness are different for bicycle and handgrip exercises, the effect of sidedness was tested separately in the subgroup analysis for both the types of exercise. A comparison of actual versus sham trials was done only for coherence, the primary outcome variable of interest in this study. For all the binary comparisons, the t‐statistic returned from the mixed model was used to estimate the *p*‐value. For multiple comparisons, *glht* function of the *Multcomp* (Hothorn et al., 2008) package was used, with Helmert's contrasts, and the *p*‐values were adjusted by Bonferroni's method. Helmert's contrast was used in two models, that is, the effect of exercise type on outcome measures and the effect of sidedness variable on the outcome measures in the subgroup analysis of bicycle exercise. As there are three levels in both the predictor variables (type of exercise: baseline, bicycle exercise, and handgrip exercise; sidedness of bicycle exercise: left side, right side, and bilateral), two Helmert's contrasts were defined for each. For the type of exercise variable, the first contrast was any exercise versus baseline, and the second, was handgrip versus bicycle exercise. For the sidedness variable, the first contrast was bilateral versus unilateral exercise, and the second, left versus right‐sided exercise. We also report the effect sizes of all the comparisons tested as *t*−/*z*‐statistic values. The details of the mixed models tested are listed in Table S1.

## 3 RESULTS

Cardiorespiratory coherence for each experimental condition is shown in Figure 2. It can be seen that coherence attains maximum value in the high‐frequency region (0.15–0.4 Hz), corresponding to the respiratory frequency. The median heart rate and respiratory rate in the anticipation period of all the exercises are shown in Figures S5 and S6.

### 3.1 Effect of the type of exercise

The mean and standard deviation values of all the metrics characterizing the type and intensity of exercise are shown in Table 2. All the participants were self‐reported to be right‐handed.

**TABLE 2:** Coherence and HRV parameters by types and intensities of exercise: The mean (SD) of coherence, HFnu and HRV parameters are shown. While coherence and HFnu decreased with expectation of any exercise and high intensity exercise, LF/HF increased concurrently

Variable	Levels	Coherence	logTP	HFnu	LF/HF
Type of exercise	Baseline	0.54 (0.16)	7.33 (1.00)	33.77 (17.48)	3.07 (2.67)
	Bicycle exercise	0.41 (0.12)	7.76 (0.86)	27.24 (14.29)	4.06 (3.40)
	Hand grip exercise	0.39 (0.12)	7.94 (0.91)	25.89 (13.51)	4.13 (3.45)
Intensity of exercise	Low intensity	0.42 (0.11)	7.80 (0.87)	28.44 (14.91)	3.85 (3.55)
	High intensity	0.37 (0.10)	7.86 (0.91)	24.96 (12.79)	4.32 (3.28)

**FIGURE 3:**
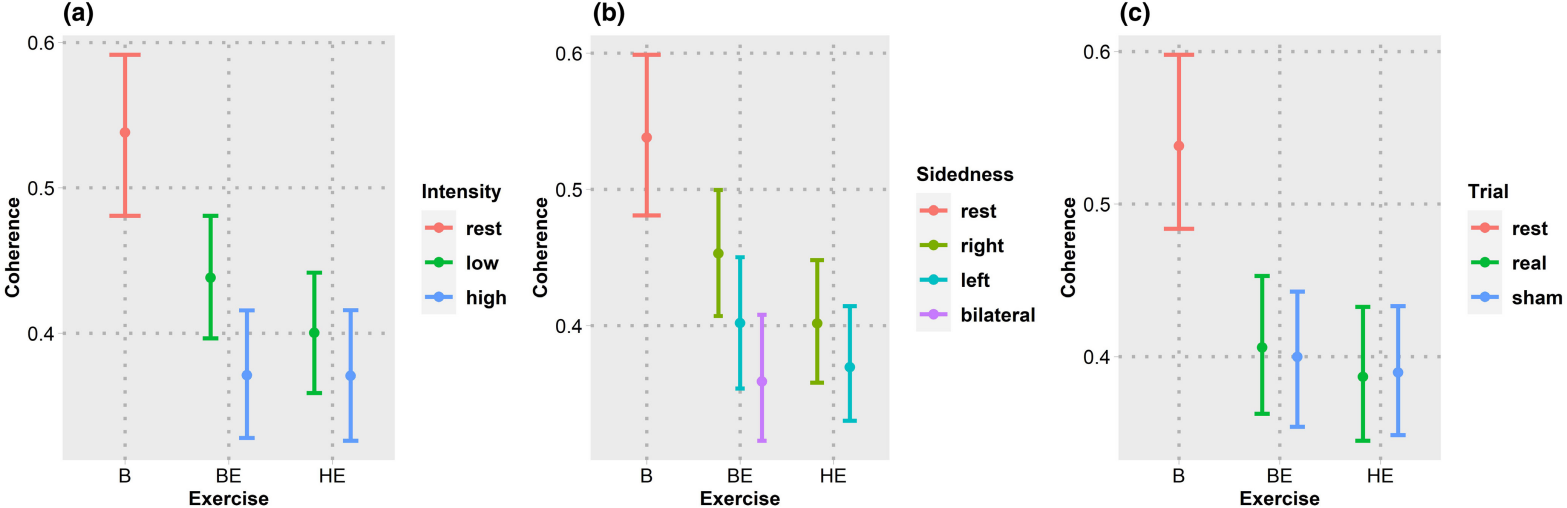
Coherence. Coherence was averaged over type, intensity, sidedness, and type of trial. Width of the error bars represent 95% confidence intervals estimated using the bootstrap method. (a–c) Coherence changes in each type exercise with the effect of intensity (a), sidedness (b), and trials (c). B, baseline; BE, bicycle exercise; HE, handgrip exercise. The fall in coherence with various exercise conditions can be seen.

**FIGURE 4:**
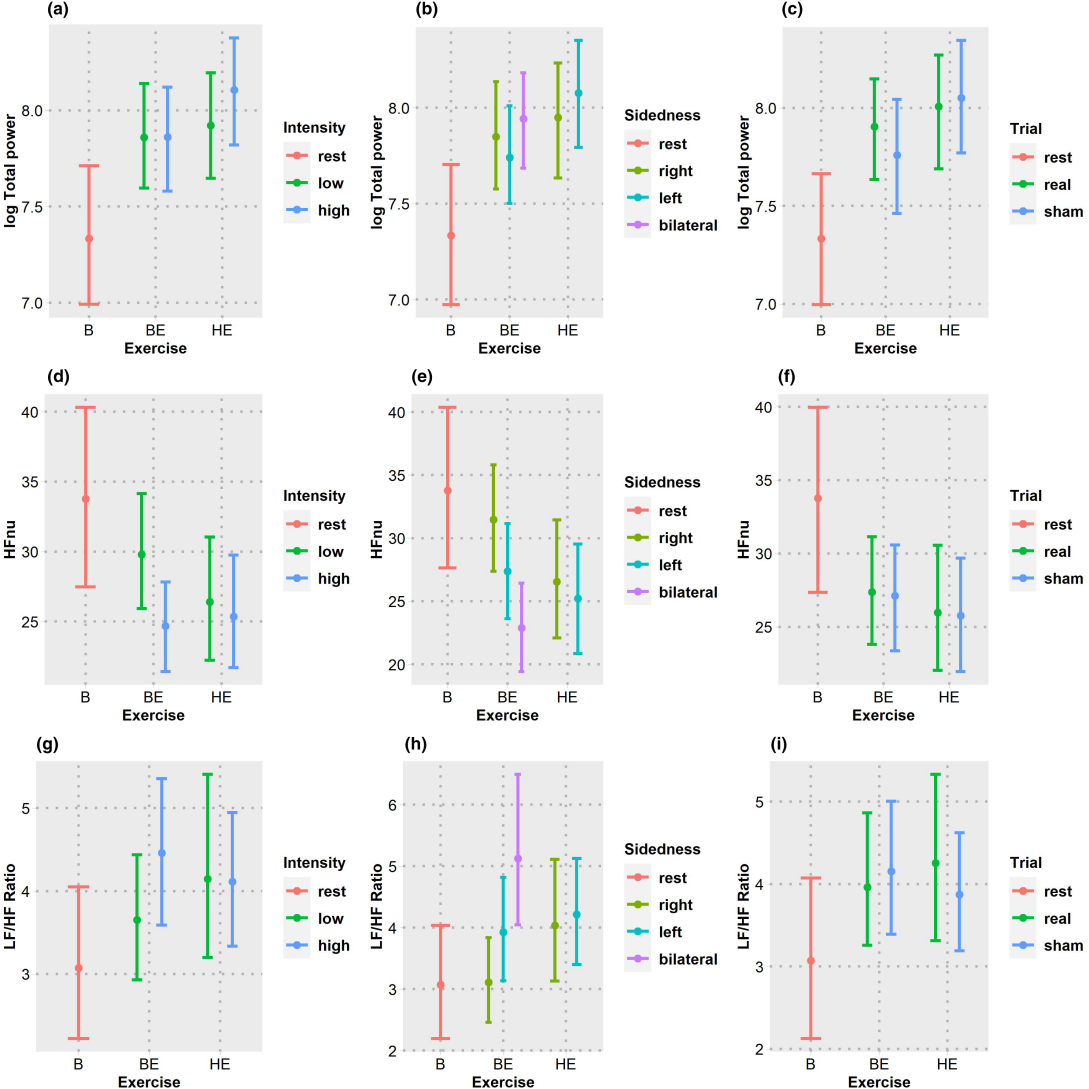
Frequency domain HRV parameters. The HRV parameters were averaged over type, intensity, sidedness and type of trial. Width of the error bars represent 95% confidence intervals estimated using bootstrap method. (a–c): log Total power changes in each type exercise with the effect of intensity (a), sidedness (b), and trials (c). (d–f) HFnu changes in each type exercise with the effect of intensity (d), sidedness (e), and trials (f). (g–i) LF/HF ratio changes in each type exercise with the effect of intensity (g), sidedness (h), and trials (i). B, baseline; BE, bicycle ergometer exercise; HE, hand grip exercise. Increase in total power and LF/HF ratio across the various exercise conditions can be seen. It can be observed that HFnu decreased concomitantly with the increase of total power and LF/HF ratio.

The coherence decreased during the anticipation period compared to that at baseline (*z* = −6.76, *p* = 4.5 × 10^−11^). HRV parameters revealed an increase in logTP (*z* = 6.73, *p* = 5.61 × 10^−10^), LF/HF ratio (*z* = 3.32, *p* = 0.0027), and a decrease in HFnu (*z* = −2.40, *p* = 0.000218). Coherence during anticipation of bicycle exercise was not found to be different from that of handgrip exercise (*z* = −0.78, *p* = 1) with a similar trend in logTP (*z* = 1.43, *p* = 0.459), HFnu (*z* = −0.65, *p* = 1), and LF/HF ratio (*z* = 0.20, *p* = 1). The trends in coherence are shown in Figure 3 and those of HRV in Figure 4.

### 3.2 Effect of the intensity of exercise

Analysis of complete dataset revealed that there was a decrease in coherence (*t* = −8.69, *p* = 9.7 × 10^−10^) and HFnu (*t* = −4.00, *p* = 0.0004) while there was increase in logTP (*t* = 2.19, *p* = 0.0367), LF/HF ratio (*t* = 2.26, *p* = 0.0315).

The mean and standard deviation values of the metrics characterizing the effect of the exercise intensity are shown in Table 3.

**TABLE 3:** Mean (SD) of coherence and HRV parameters by type and intensity of exercise—sub‐group analysis for effect of intensity: sub‐group analysis was done by computing mean (SD) for low and high intensity exercise separately for each type of exercise. Fall of coherence with intensity is seen in both bicycle and handgrip exercise with a greater fall associated with the bicycle exercise

Exercise	Parameter	Coherence	logTP	HFnu	LF/HF
Bicycle ergometer exercise	Low intensity	0.44 (0.12)	7.78 (0.83)	29.80 (15.26)	3.65 (3.30)
	High intensity	0.37 (0.12)	7.75 (0.89)	24.69 (12.77)	4.46 (3.47)
Hand grip exercise	Low intensity	0.40 (0.12)	7.84 (0.92)	26.41 (14.17)	4.14 (3.88)
	High intensity	0.37 (0.12)	8.04 (0.91)	25.36 (12.84)	4.11 (2.96)

In the subgroup analysis for bicycle exercise, coherence was found to decrease (*t* = −8.15, *p* = 3.57 × 10^−9^) with an increase in exercise intensity. There was a decrease in HFnu (*t* = −3.89, *p* = 0.0006) and an increase in LF/HF ratio (*t* = 3.68, *p* = 0.001) with the anticipation of high‐intensity exercise. LogTP did not change (*t* = 0.02, *p* = 0.9826) with intensity of exercise.

Subgroup analysis for handgrip exercise revealed a similar fall in coherence (*t* = −3.58, *p* = 0.0013) with increase in intensity of exercise. LogTP (*t* = 3.40, *p* = 0.002) increased with intensity while HFnu (*t* = −1.32, *p* = 0.1965) and LF/HF ratio (*t* = −0.01, *p* = 0.9318) did not change significantly.

The effect of intensity interacted with that of the type of exercise. The fall of coherence with increase in intensity was greater for bicycle exercise when compared to handgrip exercise. (*z* = 3.27, *p* = 0.00433). HRV parameters that revealed a significant interaction between exercise and intensity are logTP (*z* = 2.52, *p* = 0.0476) and, HFnu (*z* = 2.70, *p* = 0.0281). LF/HF ratio did not show any interaction effect (*z* = −1.98, *p* = 0.1898).

### 3.3 Effect of the side of exercise

The mean and standard deviation values of the metrics characterizing the effect of the side of exercise are shown in Table 4.

**TABLE 4:** Mean (SD) of coherence and HRV parameters by type and side of exercise‐sub‐group analysis for effect of sidedness: sub‐group analysis was done by computing mean (SD) for left, right and bilateral exercise for bicycle exercise; left and right sided exercise for handgrip exercise coherence was lesser for bilateral when compared to unilateral exercise for the bicycle exercise. Coherence was less for left sided when compared to right sided exercise for both bicycling and handgrips

Exercise	Side of exercise	Coherence	logTP	HFnu	LF/HF
Bicycle exercise	Right sided exercise	0.45 (0.16)	7.78 (0.89)	31.46 (14.64)	3.11 (2.40)
	Left sided exercise	0.40 (0.16)	7.65 (0.85)	27.38 (13.77)	3.93 (3.10)
	Bilateral exercise	0.36 (0.15)	7.86 (0.83)	22.89 (13.18)	5.13 (4.18)
Hand grip exercise	Right sided exercise	0.40 (0.15)	7.86 (0.97)	26.56 (13.84)	4.04 (3.51)
	Left sided exercise	0.37 (0.14)	8.02 (0.86)	25.22 (13.84)	4.22 (3.40)

For the bicycle exercise, anticipation period coherence was substantially less for bilateral exercise when compared to unilateral exercise (*z* = −4.58, *p* = 1.37 × 10^−5^). Among the HRV parameters, logTP (*z* = 2.63, *p* = 0.0255) and LF/HF (*z* = 4.26, *p* = 6.12 × 10^−5^) were higher, and HFnu (*z* = −4.86, *p* = 3.53 × 10^−6^) was lower for bilateral exercise when compared to the unilateral exercise. In the unilateral exercise, coherence (*z* = −2.96, *p* = 0.00925) and HFnu (*z* = −4.86, *p* = 3.53 × 10^−6^) were lesser for left‐sided exercise when compared to the right‐sided exercise. There was no significant difference between left‐ and right‐sided exercise for logTP (*z* = −1.64, *p* = 0.3003), LF/HF ratio (*z* = 1.88, *p* = 0.182).

Subgroup analysis for handgrip exercise revealed a trend in coherence similar to that of bicycle exercise, i.e., coherence for anticipation of left‐sided exercise was lower than that of right‐sided exercise (*t* = −3.00, *p* = 0.0056). HRV parameters did not show significant differences between left‐ and right‐sided exercise (logTP: *t* = 2.02, *p* = 0.0529|HFnu: *t* = −1.14, *p* = 0.2661|LF/HF: *t* = 0.61, *p* = 0.5489).

### 3.4 Effect of the type of trial

Coherence in actual and sham trials did not reveal any significant differences across various exercise conditions. The results are summarized in Table S2.

### 3.5 Surrogate data analysis

Surrogate data analysis was performed by computing coherence between randomly shuffled heart rate series and resampled‐interpolated respiratory rhythm time series, as shown in Figure 2. Visual inspection of the plot revealed a drastically decreased coherence compared to the same without random shuffle, indicating that the Coherence metric calculated from the data is not due to a random correlation between two‐time series.

## 4 DISCUSSION

In this study, we have explored the effect of exercise anticipation on cardiorespiratory coherence, a measure of RSA. We have shown that anticipation of physical exercise decreases the coherence between heart rate and respiratory rhythm compared to baseline. We found that this decrease in coherence depends on the expected intensity and the muscle mass. The expectation of high‐intensity exercise had a greater drop in coherence when compared to the low‐intensity one, irrespective of the type of exercise. Greater the anticipated muscle mass, more was the fall of coherence, as bilateral bicycle exercise had lower coherence when compared to the unilateral (right‐sided or left‐sided) exercise. We also observed a greater fall of coherence with left‐sided than right‐sided exercise, for bicycle and handgrip exercises. Although we did not find a significant difference in the anticipatory coherence between the two types of exercise, they differed in the effect of intensity. There was a greater fall of coherence in anticipation of increased intensity with bicycle exercise than with handgrip exercise.

In contrast to the effects on coherence, the response of HRV parameters was variable. HFnu, a normalized measure of respiratory influence on heart rhythm, decreased with exercise expectation. This decrease covaried with the expected intensity and muscle mass. The overall heart rate variability, captured by the logTP, increased with the anticipation of exercise and scaled with expected intensity and muscle mass. LF/HF ratio rose in parallel with the fall of coherence with most exercise‐related conditions except for the handgrip exercise, suggesting alteration of sympathovagal balance consistent with increased sympathetic modulation due to expectation of activity. Effect of sidedness was weaker for all HRV parameters. Hence, in response to the cognitive load imposed by anticipation, the trends related to altered cardiorespiratory dynamics appear stronger with coherence than HRV metrics.

In the context of the discussion above, it is helpful to compare and contrast cardiorespiratory coherence and HFnu as measures of RSA. Coherence is a less variable metric, as evidenced by lower values of coefficient of variation (CV = standard deviation/mean) compared to HFnu (CV values are reported in Tables S3–S5). The effect sizes of coherence are, in general, more prominent than those of HFnu. We have reported standardized effect size metrics like *t*‐statistic/*z*‐statistic values for every comparison made. Except for the effect of sidedness in bicycle exercise, coherence has larger *t*‐/*z*‐statistic values than HFnu. As a result, we infer that coherence can detect subtler differences between experimental conditions compared to HRV metrics, including HFnu. For example, the difference between left‐ and right‐sided handgrip exercises was statistically significant for coherence, unlike the null result with HFnu. Moreover, by including respiratory signal in the computation of coherence, the estimate obtained is enriched in respiratory modulation‐related effects. In contrast, HFnu reflects summated vagal action that includes non‐respiratory and respiratory components. Therefore, we suggest that coherence may be a more robust and physiologically plausible metric of RSA than HFnu.

We propose two mutually non‐exclusive mechanisms to explain the results discussed above: (i) stress/anxiety induced vagal withdrawal in the pre‐movement anticipation period and (ii) cardiorespiratory‐autonomic control processes that vary qualitatively for different movement patterns. The decrease in vagal activity during stress associated with various mental and motor tasks is well known. Cognitive load associated with mental attention, working memory tasks (Muth et al., 2012; Overbeek et al., 2014), and anticipation of stressful situations (Pulopulos et al., 2018) have been found to cause a decrease in RSA. An increase in sympathetic activity or LF/HF has also been reported in identical experimental conditions (Freyschuss, 1970; Krogh & Lindhard, 1913; Obrist, 1981; Thornton et al., 2001). Similar changes in HF and LF/HF ratio were shown in the pre‐movement phase of various activities like swimming, biking, and engaging with movement simulators and were also found to be associated with anxiety/awareness ratings (Cervantes Blásquez et al., 2009; Mateo et al., 2012; Saus et al., 2006, 2012). Most of the above studies were simple paradigms that explored the difference in HRV between baseline and pre‐movement phase of one or two activities. Whether anxiety levels alone can explain the intricate covariance between various exercise‐related variables and coherence/HRV obtained in the present study must be explored in future studies.

Static and dynamic exercises have been shown to elicit a different pattern of autonomic response, suggesting that cardiorespiratory‐autonomic control processes are differentially organized based on the task attributes and requirements (González‐Camarena et al., 2000). In the study cited above, static exercise was found to have a higher vagal tone during movement than dynamic exercise. Vasopressor response during static exercise is thought to trigger a baroreflex‐induced increase in vagal tone. We found concordant results with our premovement data wherein fall of coherence and HFnu was greater with bicycle exercise (dynamic) than handgrip exercise (static exercise). Based on the literature and our findings on motor task‐dependent autonomic response patterns, we conjecture that autonomic responses during anticipation are yoked to the motor planning processes implicit in internal models of movement. Obrist proposed the “cardiac‐somatic hypothesis”, according to which somatomotor response and autonomic response are integral parts of a more globally integrated response. Such responses are organized by the higher centers during voluntary movements and released as a “coordinated set,” even if the somatomotor component is suppressed from release, by instruction or pharmacological blockade (Obrist, 1981; Obrist et al., 1969, 1970a, 1970b, 1974). The present study instructed the participant to “withhold” the movement in the anticipation period until after the second cue. Nevertheless, the coherence is reduced in anticipation of exercise, similar to what is known to occur with RSA in mild–moderate physical exercise.

Another interesting finding is the decrease in coherence with the anticipation of left‐sided exercise compared to right‐sided exercise. Ahern et al. (2001) noted that intracarotid injections of sodium amytal into the right side evoked larger and faster changes in the heart rate than on the left side. Asymmetries have also been documented in the innervation of the heart. SA node of the heart receives innervation predominantly from the right‐sided sympathetic and parasympathetic nervous systems (Schwartz, 1984). Craig (2005), on the other hand, proposed a different idea of autonomic asymmetry where the left cerebral hemisphere controls parasympathetic‐vagal modulation and the right cerebral hemisphere controls sympathetic modulation of visceromotor function. In the context of exercise anticipation, data from the present study support right cerebral hemispheric control of vagal modulation. The cardiorespiratory coherence in the high‐frequency band appears to decrease more—with left‐sided exercise, initiated by the right side of the brain. Although we did not use any structured handedness testing inventory to assess hand preference of participants, all of them were self‐reported to be right‐handed. Whether the left–right asymmetry we have shown can be attributed to invariable asymmetry in autonomic control, dominance related to handedness or differential stress/anxiety levels could be explored in further studies.

**FIGURE 5:**
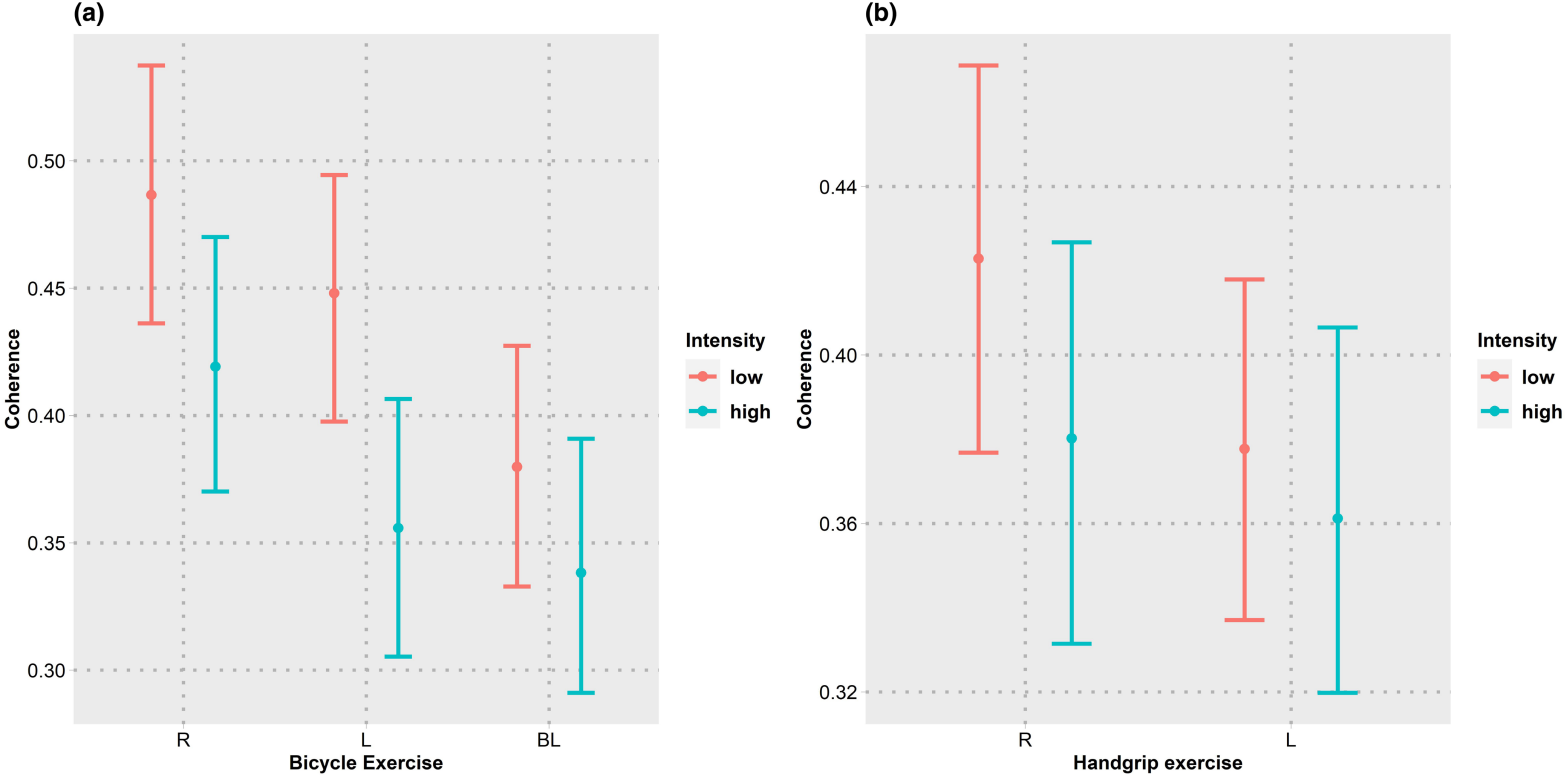
Carryover effects on coherence. (a) Bicycle exercise, (b) handgrip exercise. Width of the error bars represent 95% confidence intervals using the bootstrap method. No obvious linear trend over the order in which exercises were done for each exercise type, intensity, muscle mass, and sidedness, stands out prominently, indicating that carryover bias may not be a potential cause of trends observed.

The study has the following limitations. The order of 10 different exercises was not randomized, raising the possibility of carryover bias and confounding by learning effects. We believe that we minimized the influence of carryover bias by ensuring an intervening period of rest until the heart rate returns to and is maintained within the resting state range of heart rate for 1 min. Moreover, we empirically investigated the effect of carryover by subgroup plots for bicycle and handgrip exercises to look for a linear trend, possibly engendered by the carryover bias, along with the order in which various exercises were performed, and no such trend was found, as shown in Figure 5. Therefore, carryover effects could be potentially ruled out as a cause of trends in the present study. Practice sessions before the experiment minimized the confounding due to learning. Only males were included in the study affecting the generalizability of our findings. Due to the lack of left‐handed participants in our cohort, we cannot disambiguate the two possible explanations for asymmetry in coherence effects with side of exercise viz., Dominance related to handedness or fixed asymmetry in autonomic control.

## 5 IMPLICATIONS OF THE STUDY

The results of our research also have clinically relevant implications for patients with ventilation‐perfusion mismatch and ischemic heart disease. As mentioned previously, RSA has been hypothesized to improve gas exchange efficiency by optimizing the ventilation‐perfusion matching in the lungs (Hayano & Yasuma, 2003) and minimizing the workload on the heart while maintaining blood gas homeostasis (Ben‐Tal et al., 2012). Diminished RSA in the expectation of exercise may have adverse clinical consequences in these patient cohorts. As graded physical activity is an integral component of rehabilitation in many heart and lung diseases, clinical implications of pre‐exercise fall in RSA warrant further investigation. As the changes in the pre‐movement phase are similar to those during activity, dynamics of anticipatory coherence can offer valuable insights to cardiorespiratory‐autonomic control processes that transpire during movement while avoiding the problem of artifacts induced by motion. In sports physiology, pre‐exercise coherence can be explored to predict subsequent motor performance and training effects.

## 6 CONCLUSION

In conclusion, the anticipation of exercise results in cardiorespiratory coherence changes, similar to those during the actual exercise. We found that mere anticipation of exercise leads to a fall in coherence in the high‐frequency band, the frequency region of RSA. Coherence decreased with the expectation of higher intensity and bilateral exercise, while in the unilateral exercise, it was lower for left‐sided exercise. The results suggest that the cardiorespiratory‐autonomic control mechanisms in the pre‐movement phase are task‐dependent and enable the prediction of subsequent motor performance. Alternatively, premovement stress can account in part for the changes we report. Scaling of coherence with various exercise‐related variables during anticipation suggests that cardiorespiratory‐autonomic control is yoked to the motor system in multidimensional internal models, the coordinated action of which might prepare the body for the phase transition from resting to exercise state.

## AUTHOR CONTRIBUTIONS

Aditya Koppula: Conceptualization, Methodology, Investigation, Formal analysis, Data curation, Writing‐Original draft, Writing‐Review & Editing, Visualization, Project administration, Funding acquisition. Ram Reddy Barra: Conceptualization, Methodology, Writing‐Review & Editing, Supervision. Kousik Sarathy Sridharan: Formal analysis, Data curation, Writing‐Original draft, Writing‐Review & Editing, Visualization.

## FUNDING INFORMATION

This work was funded by an intramural grant provided by Multidisciplinary Research Unit‐Gandhi Medical College (MDRU), where the study was conducted.

## CONFLICT OF INTEREST

The authors declare that the research was conducted in the absence of any commercial or financial relationships that could be construed as a potential conflict of interest.

## ETHICS STATEMENT

The study was approved by the Institutional Ethical Committee, Gandhi Medical College (Rc.No.IEC/ GMC/2017/XXXVIII). The study conformed to the standards set by the Declaration of Helsinki.

## Supporting information


Table S1
Table S2
Table S3
Table S4
Table S5
Figure S1
Figure S2
Figure S3
Figure S4
Figure S5
Figure S6
