# Supplementary material for: Effects of exercise anticipation on cardiorespiratory coherence
Source: Physiol Rep. 2022 Jul 26;10(14):e15381. doi: 10.14814/phy2.15381 (PMC9325975; doi:10.14814/phy2.15381)
Supplement: Supplementary file 1 — Table S1 Table S2 Table S3 Table S4 Table S5 Figure S1 Figure S2 Figure S3 Figure S4 Figure S5 Figure S6 [file PHY2-10-e15381-s001.docx]

**Supplementary information**

**Table S1: Details of mixed models:** Outcome variables tested, level at which the data aggregation was done, predictor variables for the model and the details of model formulation are mentioned. In the model formula, fixed effects are denoted by the term “fixed” and random effects by “random” in the model formulae. Predictor variables are included as fixed effects, and the also as random effects. Repeated measures nature of the same is accounted for by nesting within the individual participant, denoted by participant ID, in the random effects.

| Outcome variables | Data analysis level | Predictor variable | Model |
| --- | --- | --- | --- |
| Coherence, logTP, HFnu, LF/HF ratio, | Entire Data | Type of exercise | lme(fixed=outcome~type of exercise, random= ~1\|Participant ID/type of exercise) |
|  |  | Intensity | lme(fixed=outcome~Intensity, random= ~1\|Participant ID/Intensity) |
|  |  | Interaction (Type of exercise *Intensity) | lme(fixed=outcome~type of exercise*Intensity, random= ~1\|Participant ID/type of exercise/Intensity) |
|  | Subgroup analysis: Bicycle exercise (BE) | Intensity | lme(fixed=outcome~Intensity, random= ~1\|Participant ID/Intensity, data=BE) |
|  |  | Sidedness | lme(fixed=outcome~Intensity, random= ~1\|Participant ID/Sidedness, data=BE) |
|  | Subgroup analysis: Handgrip exercise(H) | Intensity | lme(fixed=outcome~Intensity, random= ~1\|Participant ID/Intensity, data=H) |
|  |  | sidedness | lme(fixed=outcome~Intensity, random= ~1\|Participant ID/Sidedness, data=H) |
| Coherence |  | Trial | lme(fixed=outcome~Trial, random= ~1\|Participant ID/Trial) |

**Table S2: Comparison of Coherence in real vs sham trial comparisons across exercise conditions:** Table shows subset of data for which the comparison was made (condition), summary statistics for real and sham trials in the form of Mean (SD) and the results of linear mixed model for the comparison, shown as t-statistic (p-value). It is observed that there is no significant difference between real trials and sham trials across the various exercise conditions.

| S. No | Condition | Real trials  Mean (SD) | Sham trials  Mean (SD) | | t-statistic (p-value) |
| --- | --- | --- | --- | --- | --- |
| 1 | Overall data | 0.40 (0.11) | | 0.39 (0.10) | t=**-** 0.62 (p=0.543) |
| 2 | Bicycle exercise | 0.41 (0.13) | | 0.40 (0.12) | t=**-** 0.52 (p=0.6084) |
| 3 | Hand grip exercise | 0.39 (0.12) | | 0.39 (0.12) | t= 0.30 (p=0.7656) |
| 4 | Hand grip exercise-Low intensity- Left side | 0.38 (0.12) | | 0.35 (0.14) | t=**-** 1.14 (p=0.2668) |
| 5 | Hand grip exercise-Low intensity - Right side | 0.42 (0.14) | | 0.44 (0.11) | t= 0.63 (p=0.5396) |
| 6 | Hand grip exercise-High intensity- Left side | 0.36 (0.15) | | 0.38 (0.13) | t= 0.48 (p=0.6331) |
| 7 | Hand grip exercise-High intensity - Right side | 0.37 (0.14) | | 0.39 (0.134) | t= 1.12 (p=0.2319) |
| 8 | Bicycle exercise-Low intensity-Left side | 0.45 (0.15) | | 0.47 (0.18) | t= 0.91 (p=0.375) |
| 9 | Bicycle exercise-Low intensity-Right side | 0.48 (0.15) | | 0.50 (0.14) | t= 0.87 (p=0.3985) |
| 10 | Bicycle exercise-High intensity-Left side | 0.36 (0.15) | | 0.32 (0.12) | t=**-** 1.25 (p=0.2251) |
| 11 | Bicycle exercise-High intensity-Right side | 0.43 (0.16) | | 0.41 (0.15) | t=**-** 0.91 (p=0.3716) |
| 12 | Bicycle exercise-Low intensity-Bilateral | 0.37 (0.14) | | 0.41 (0.15) | t= 1.41 (p=0.1752) |
| 13 | Bicycle exercise-High intensity-Bilateral | 0.33 (0.14) | | 0.35 (0.15) | t= 1.41 (p=0.1752) |

It can be seen from Tables S3-5, the CV is smaller for coherence when compared to HFnu. This indicates, as measure of Respiratory sinus arrhythmia (RSA), coherence is less variable than HFnu.

**Table S3: Coefficient of variation (CV) for Coherence and HFnu by types and intensities of exercise:** Coefficient of variation (CV=Standard deviation/mean)

| Variable | Levels | Coherence | HFnu |
| --- | --- | --- | --- |
| Type of Exercise | Baseline | 0.3 | 0.52 |
|  | Bicycle exercise | 0.29 | 0.52 |
|  | Hand Grip exercise | 0.31 | 0.52 |
| Intensity of Exercise | Low intensity | 0.26 | 0.52 |
|  | High intensity | 0.27 | 0.51 |

**Table S4: Coefficient of variation (CV) for Coherence and HFnu by types and intensities of exercise- Sub-group analysis for effect of intensity:** Coefficient of variation (CV=Standard deviation/mean)

| Exercise | Parameter | Coherence | HFnu |
| --- | --- | --- | --- |
| Bicycle Ergometer exercise | Low intensity | 0.27 | 0.51 |
|  | High intensity | 0.32 | 0.52 |
| Hand Grip exercise | Low intensity | 0.3 | 0.54 |
|  | High intensity | 0.32 | 0.51 |

**Table S5: Coefficient of variation (CV) for Coherence and HFnu by type and side of exercise- Sub-group analysis for effect of sidedness:** Coefficient of variation (CV=Standard deviation/mean)

| Exercise | Side of exercise | Coherence | HFnu |
| --- | --- | --- | --- |
| Bicycle exercise | Right sided exercise | 0.36 | 0.47 |
|  | Left sided exercise | 0.4 | 0.5 |
|  | Bilateral exercise | 0.42 | 0.58 |
| Hand Grip Exercise | Right sided exercise | 0.38 | 0.52 |
|  | Left sided exercise | 0.38 | 0.55 |

**Figures:**

**Fig. S1: Pulsus trigeminus in a participant.** Every third beat is a premature ventricular contraction (PVC)/ectopic beat that comes earlier and is followed longer pause before next beat occurs. The morphology of the PVC is abnormal with a QRS complex of longer duration.


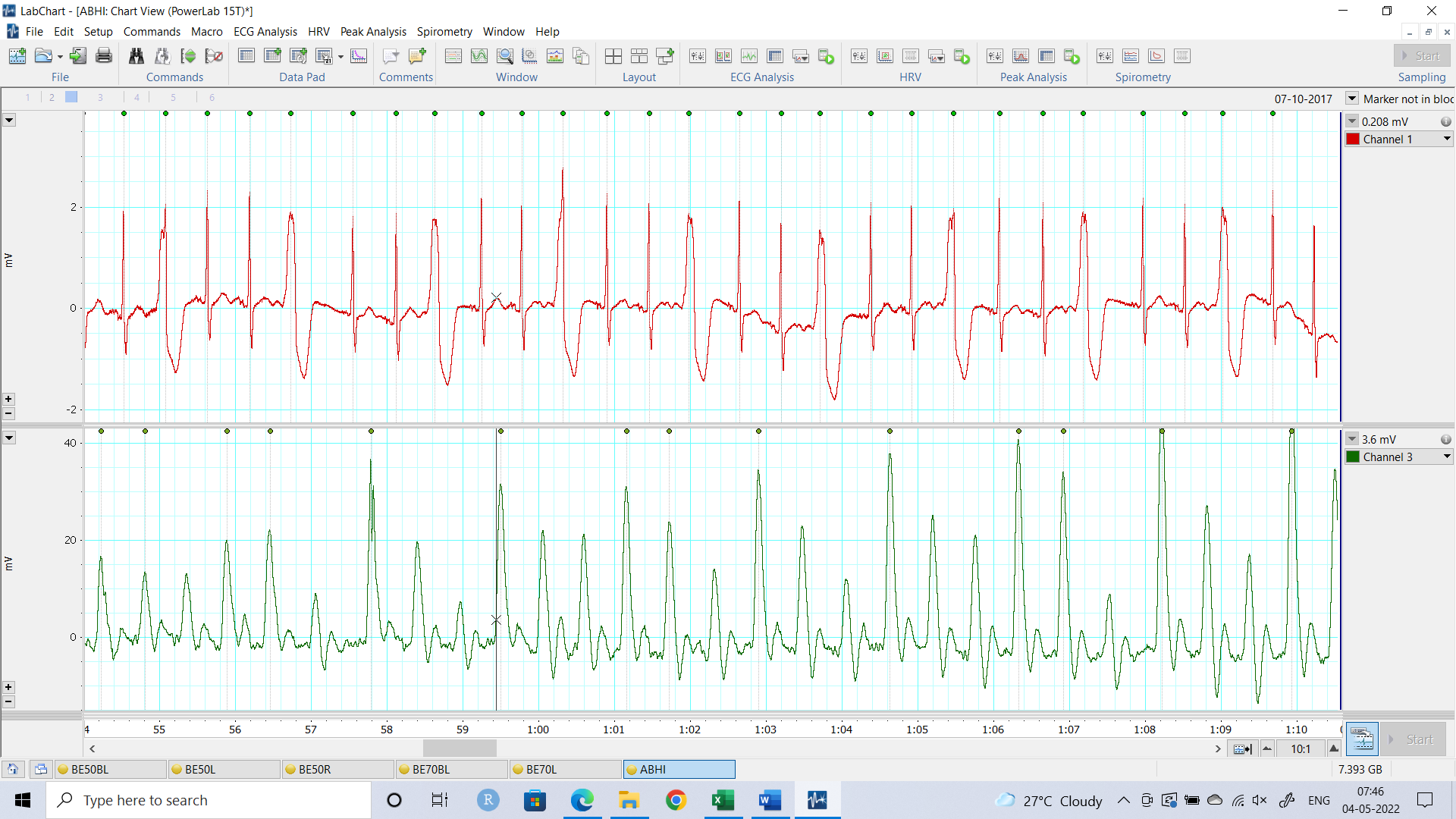


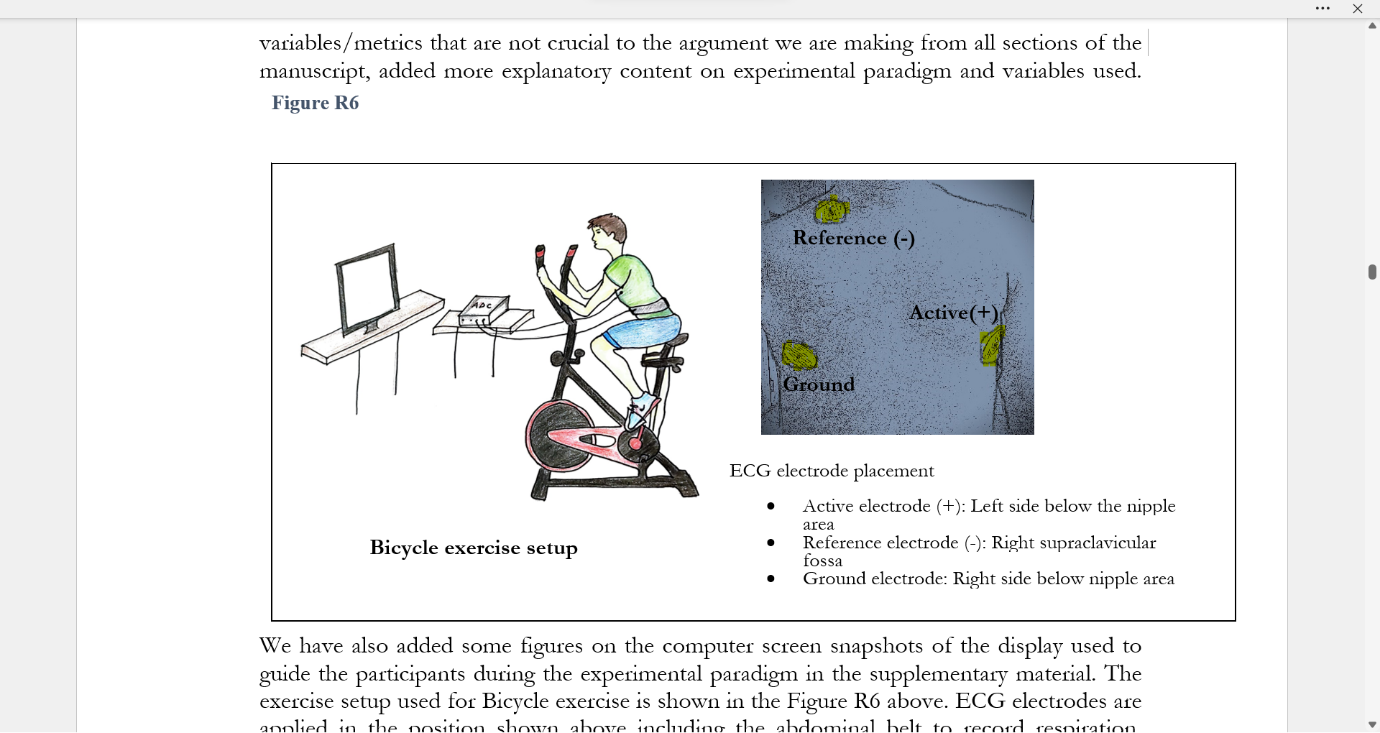
**Fig. S2. Bicycle exercise setup and ECG electrode placement.**

**Fig. S3. Visual feedback during bicycle exercise.** Top panel is instantaneous heart rate with a green zone marking the target heart rate. Participant is instructed exercise to push the heart closer to the red-zone, above the green-area, as soon as an auditory cue BEEP2 is played following which the exercise is done for 30 seconds. Middle panel is ECG and bottom panel is respiratory rhythm.


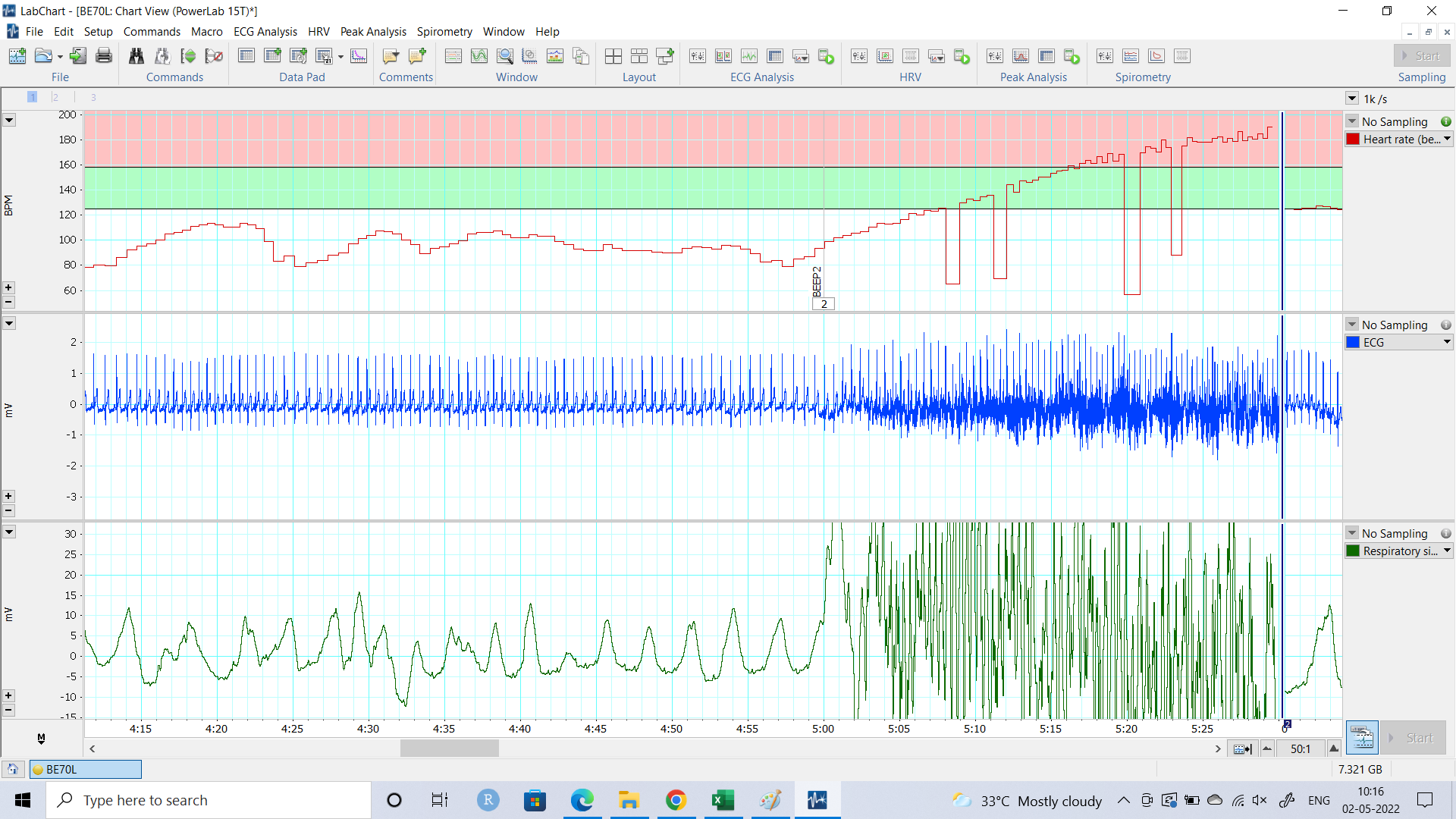


**Fig. S4*.* Visual feedback during handgrip exercise.** Top panel is the trace of instantaneous grip force (marked red) expressed as a percentage of maximal voluntary grip contraction with the cyan zone marking the target of grip force exertion. The limits of the cyan zone are 35% and 50% of maximum grip force. Participant is instructed exercise to push the grip force closer to the target line (35% or 50%), as soon as an auditory cue BEEP2 is played following which the exercise is done for 30 seconds. Middle panel is ECG and bottom panel is respiratory rhythm.


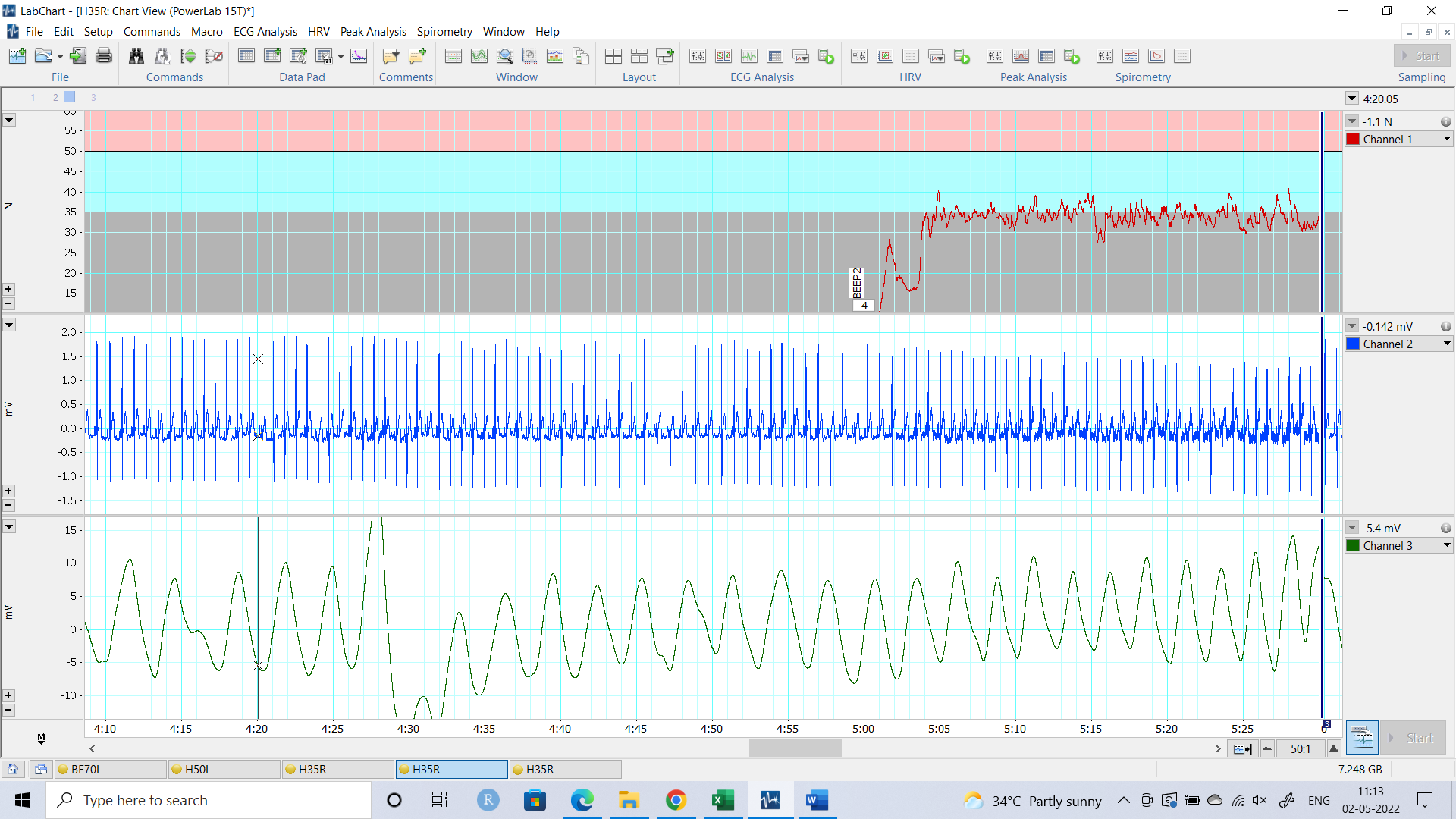


**Fig. S5. Median heart rate and respiration rate in the resting state and anticipation period of various exercises.** It can be seen that the variability of heart rate and respiratory rate during anticipation period is less than that during the baseline. This indicated the coherence decrease during anticipation is unlikely to be due increased variability due to noisy data.


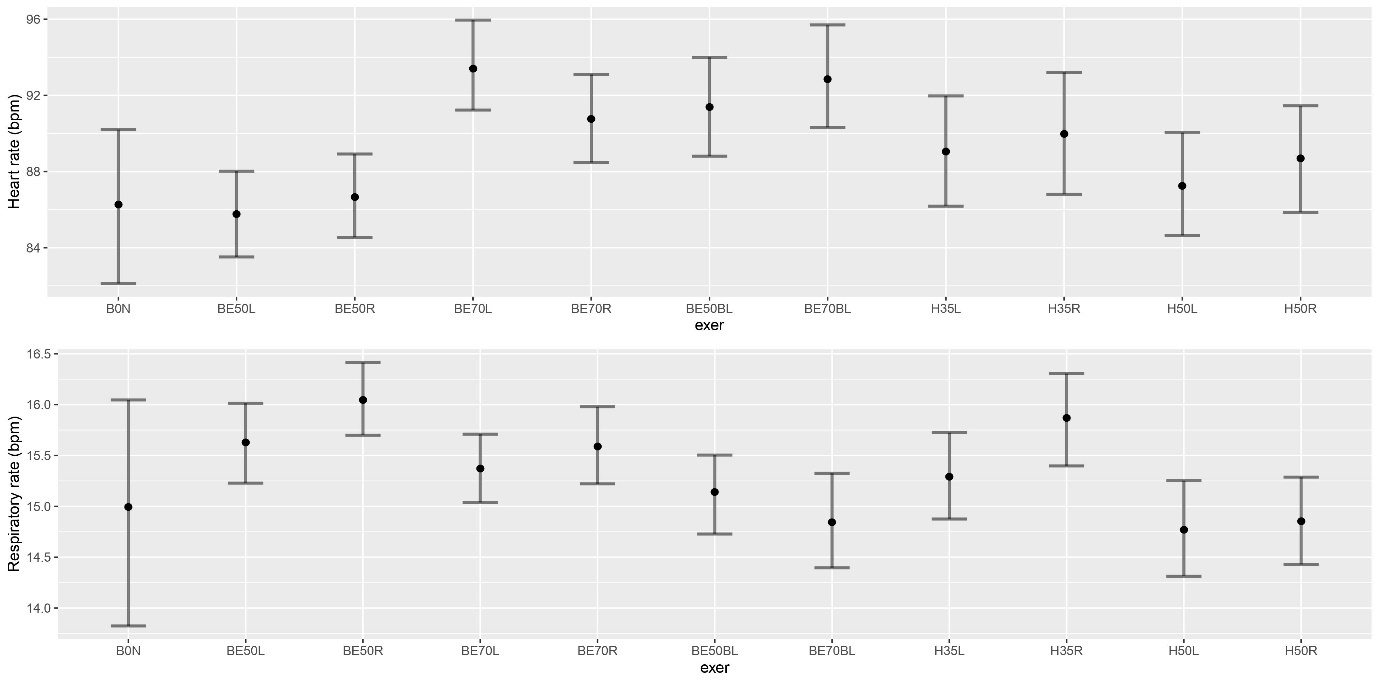


**Fig. S6. Median heart rate and Respiratory rate in the anticipation period and baseline as function of type of exercise, intensity, sidedness.**


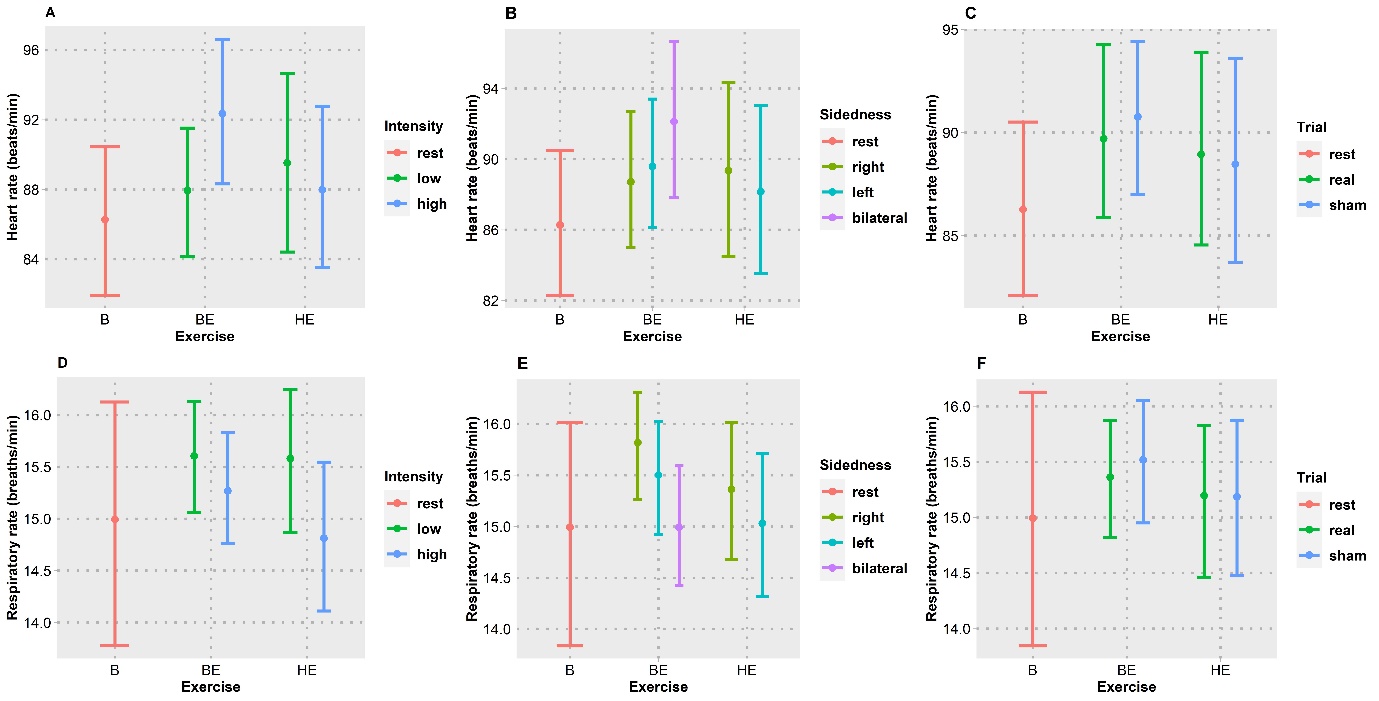


*B*=Baseline, *BE*=Bicycle exercise, *HE*=Handgrip exercise

**Acknowledgement**:

We thank Ms. JBVN Mounica, MTech student, IIT Hyderabad for her illustrative sketch of the experimental setting shown in Fig.S2
